# SIRT1 Regulates Thyroid-Stimulating Hormone Release by Enhancing PIP5Kγ Activity through Deacetylation of Specific Lysine Residues in Mammals

**DOI:** 10.1371/journal.pone.0011755

**Published:** 2010-07-23

**Authors:** Sayaka Akieda-Asai, Nobuhiro Zaima, Koji Ikegami, Tomoaki Kahyo, Ikuko Yao, Takahiro Hatanaka, Shun-ichiro Iemura, Rika Sugiyama, Takeaki Yokozeki, Yoshinobu Eishi, Morio Koike, Kyoji Ikeda, Takuya Chiba, Haruyoshi Yamaza, Isao Shimokawa, Si-Young Song, Akira Matsuno, Akiko Mizutani, Motoji Sawabe, Moses V. Chao, Masashi Tanaka, Yasunori Kanaho, Tohru Natsume, Haruhiko Sugimura, Yukari Date, Michael W. McBurney, Leonard Guarente, Mitsutoshi Setou

**Affiliations:** 1 Frontier Science Research Center, University of Miyazaki, Miyazaki, Japan; 2 Mitsubishi Kagaku Institute of Life Sciences (MITILS), Tokyo, Japan; 3 Department of Molecular Anatomy, Hamamatsu University School of Medicine, Shizuoka, Japan; 4 Department of Pathology, Hamamatsu University School of Medicine, Shizuoka, Japan; 5 Department of Medical Chemistry, Kansai Medical University, Osaka, Japan; 6 National Institute of Advanced Industrial Science and Technology, Biomedicinal Information Research Center, Tokyo, Japan; 7 Department of Physiological Chemistry, Graduate School of Comprehensive Human Sciences and Institute of Basic Medical Sciences, University of Tsukuba, Ibaraki, Japan; 8 Department of Human Pathology, Tokyo Medical and Dental University, Tokyo, Japan; 9 Department of Bone and Joint Disease, Research Institute, National Center for Geriatrics and Gerontology, Aichi, Japan; 10 Department of Investigative Pathology, Nagasaki University Graduate School of Biomedical Sciences, Nagasaki, Japan; 11 Section of Pediatric Dentistry, Division of Oral Health, Growth and Development, Faculty of Dental Science, Kyushu University, Fukuoka, Japan; 12 Department of Neurosurgery, Teikyo University Chica Medical Center, Chiba, Japan; 13 Basic Medical Science and Molecular Medicine, Tokai University School of Medicine, Kanagawa, Japan; 14 Department of Pathology and Bioresource Center for Geriatric Research, Tokyo Metropolitan Geriatric Hospital and Institute of Gerontology, Tokyo, Japan; 15 Molecular Neurobiology Program, Skirball Institute of Biomolecular Medicine, New York University School of Medicine, New York, New York, United States of America; 16 Tokyo Metropolitan Institute of Gerontology, Tokyo, Japan; 17 Ottawa Hospital Research Institute and Department of Medicine, University of Ottawa, Ottawa, Canada; 18 Department of Biology, Massachusetts Institute of Technology, Cambridge, Massachusetts, United States of America; Roswell Park Cancer Institute, United States of America

## Abstract

**Background:**

SIRT1, a NAD-dependent deacetylase, has diverse roles in a variety of organs such as regulation of endocrine function and metabolism. However, it remains to be addressed how it regulates hormone release there.

**Methodology/Principal Findings:**

Here, we report that SIRT1 is abundantly expressed in pituitary thyrotropes and regulates thyroid hormone secretion. Manipulation of SIRT1 level revealed that SIRT1 positively regulated the exocytosis of TSH-containing granules. Using LC/MS-based interactomics, phosphatidylinositol-4-phosphate 5-kinase (PIP5K)γ was identified as a SIRT1 binding partner and deacetylation substrate. SIRT1 deacetylated two specific lysine residues (K265/K268) in PIP5Kγ and enhanced PIP5Kγ enzyme activity. SIRT1-mediated TSH secretion was abolished by PIP5Kγ knockdown. SIRT1 knockdown decreased the levels of deacetylated PIP5Kγ, PI(4,5)P_2_, and reduced the secretion of TSH from pituitary cells. These results were also observed in SIRT1-knockout mice.

**Conclusions/Significance:**

Our findings indicated that the control of TSH release by the SIRT1-PIP5Kγ pathway is important for regulating the metabolism of the whole body.

## Introduction

Sir2 (silent information regulator 2) is a NAD^+^-dependent protein deacetylase [1], [2]. In yeast, Sir2 mediates transcriptional silencing at telomeres and regulates the pace of aging [3]. In *C.elegans*, one of the Sir2 orthologues, Sir2.1, has been shown to forestall aging by acting in a *DAF-16* signaling pathway [4]. In mammals, SIRT1, the closest mammalian orthologue of Sir2, has diverse roles in a variety of organs or tissues [5], and molecular mechanisms underlying the broad SIRT1 functions are highly complicated. SIRT1 is shown to be mainly involved in the regulations of whole body metabolism and physical activity. SIRT1 promotes free fatty acid mobilization of fat from white adipose tissues by repressing peroxisome proliferator-activated receptor-γ (PPARγ), a nuclear receptor that promotes adipogenesis [6]. SIRT1 also regulates the gluconeogenic and glycolytic pathways in liver in response to fasting by interacting with and deacetylating PGC-1α, a key transcriptional regulator of glucose production [6]. SIRT1 plays other roles in stabilizing genomic DNA and proteins [7], [8].

Recently, evidence has accumulated that SIRT1 could be involved in the endocrine system. In pancreas, SIRT1 modulates serum glucose levels by regulating pancreatic insulin through repressing the expression of mitochondrial uncoupling protein 2 (UCP2) [9]. In agreement with these findings, SIRT1 knockout (KO) mice show impaired glucose-stimulated insulin secretion [10]. Neuron-specific SIRT1 KO mice display reduced growth hormone level, which results in the impairment of body growth [11]. SIRT1 transgenic mice have reduced levels of blood cholesterol and adipokines, and are more metabolically active than littermate controls [12]. SIRT1 is expressed in the anorexigenic proopiomelanocortin (POMC) neurons [13], and hypothalamic SIRT1 mediates the up-regulation of the S6K pathway [14]. Despite the accumulation of evidence for the importance of SIRT1 in endocrine system, highly basic questions have remained to be addressed such as whether SIRT1 regulates metabolism in the brain and pituitary gland.

Hormones and neurotransmitters are transported as secretory granules by diverse cellular processes. Long-distance transport of these vesicles is regulated by kinesin-driven transport [15], [16] and posttranslational modifications, such as tyrosination [17] and polyglutamylation [18]. When the vesicles come to the terminal points, the process is passed to the exocytotic mechanisms [19], [20], [21]. Phosphatidylinositol plays critical roles in this exocytotic step [22]. The phosphatidylinositol-4,5-bisphosphate (PI(4,5)P_2_) interacts with the some proteins involved in the exocytotic machinery, such as the Ca^2+^-dependent activator protein for secretion (CAPS) which is a priming factor, and synaptotagmin which is a Ca^2+^ sensor [23]. How SIRT1 is involved in the exocytosis machineries is poorly understood.

In the present study, we investigated how SIRT1 regulates hormone release. We identify the SIRT1 substrate, and provide evidence for a regulatory mechanism of SIRT1- and SIRT1 target-dependent hormone release.

## Results

### Abundant expression of SIRT1 in pituitary thyrotropes

**Figure 1:**
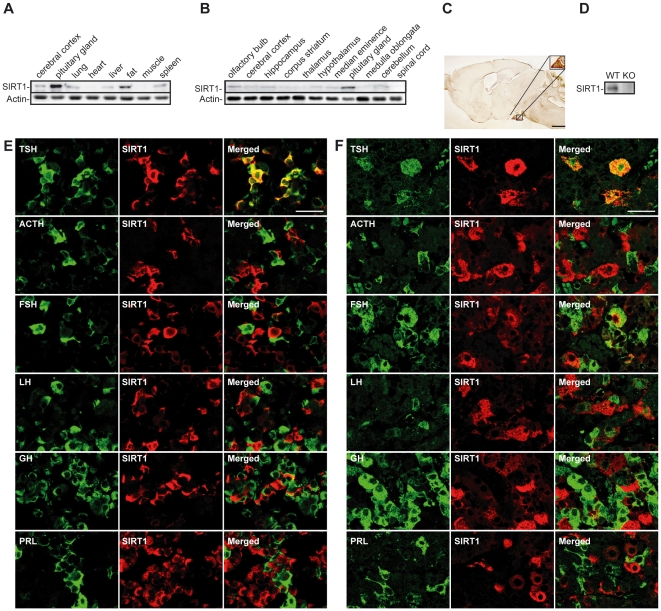
Effect of SIRT1 on hormone secretion. (A, B) Western blot analysis of SIRT1 in the systemic organs (A) and nerve tissues (B) of mice. (C) Immunohistochemical staining of SIRT1 in the rat brain. The upper right panel shows the anterior pituitary area at higher magnification. Scale bar, 1 cm. (D) SIRT1 immunoreactivity is absent in the pituitary gland of SIRT1 KO mouse. (E, F) Double immunostaining with SIRT1 (red) and pituitary hormones (green) in the pituitary gland of a 12-weeks old mouse (E) and a 64-years old human (F). Scale bar, 100 µm.

In previous work, SIRT1 KO shows the decrease in the plasma level of thyroid hormone [24]. The report suggests that SIRT1 is involved in the regulatory axis of thyroid activity or function. We thus first investigated where SIRT1 was predominantly expressed in the whole body. To this end, we performed Western blot analyses with a commercially available anti-SIRT1 antibody on lysates obtained from a variety of adult murine tissues. These assays revealed that the expression level of the SIRT1 protein in the pituitary glands was highest among that in many other adult murine tissues (Fig. 1A). The SIRT1 expression in other tissues was detected as previous reported when the expose time was elongated (data not shown). The SIRT1 expression in the pituitary gland was also highest when compared to that in other brain tissues (Fig. 1B). We next performed immunohistochemical analysis of sagittal slices of rat brain. The immunohistochemical analysis revealed strong expression of SIRT1 in the anterior pituitary (Fig. 1C). We tested the specificity of the antibody used in Fig. 1A–C by performing Western blotting with SIRT1 KO pituitary sample as a negative control (Fig. 1D). As SIRT1 signal was not detected in the SIRT1 KO tissue sample using the antibody, the antibody has an excellent specificity (Fig. 1D).

Pituitary gland is a critical component of the endocrine system that is present in all vertebrates, and pituitary hormones control homeostasis of the whole body [25]. The anterior pituitary secretes thyroid-stimulating hormone (TSH), follicle-stimulating hormone (FSH), luteinizing hormone (LH), adrenocorticotropic hormone (ACTH), growth hormone (GH), and prolactin (PRL). We used immunohistochemistry to examine the distribution of SIRT1 among hormone-secreting pituitary cells in mice and humans. SIRT1 was most frequently localized in TSH-positive cells, but barely observable in ACTH-, FSH-, LH-, GH-, and PRL-positive cells in mice (Fig. 1E) and humans (Fig. 1F). We examined human pituitary glands that were obtained during the autopsies of 20 cadavers, ranging in age from 20 to 103 years. SIRT1-positive cells were mostly found to be localized in TSH-positive cells in the case of all the cadavers (data not shown). These results show that mammalian SIRT1 is highly abundant in thyrotropes. Although normally a nuclear protein, the SIRT1 protein was in the cytoplasm of thyrotropes (Fig. 1E and 1F), a distribution similar to that reported for alpha cells of the pancreatic islets, another type of endocrine cells [9].

### SIRT1-mediated positive regulation of TSH secretion

**Figure 2:**
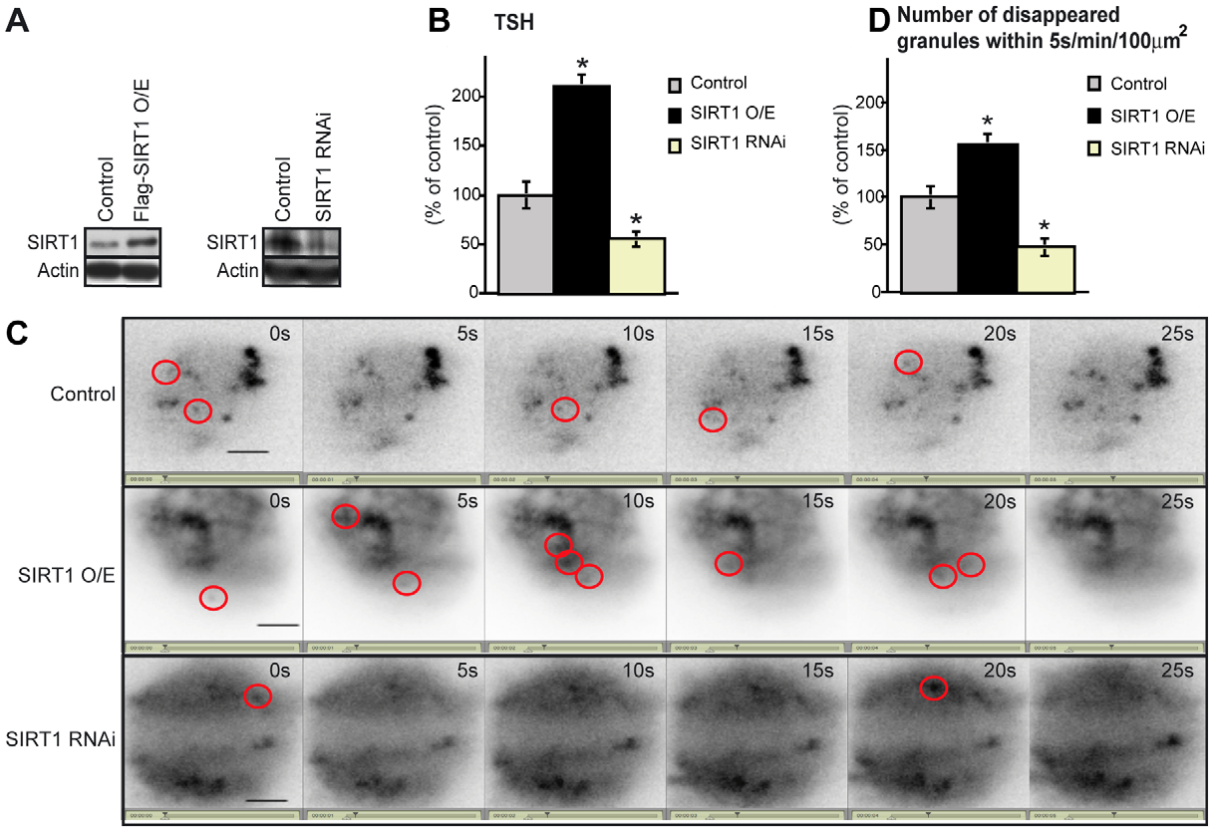
SIRT1 upregulates exocytosis of TSH. (A) Western blot analysis of SIRT1 in primary pituitary cells and in cells overexpressing (O/E) SIRT1 (left panel) and SIRT1 RNAi cells (right panel) levels. (B) Amounts of TSH secreted by SIRT1-O/E and SIRT1-RNAi pituitary cells over 24h (*n* = 10). Statistical analyses were performed using Fisher's PLSD test. *P<0.05 versus control. (C) A TIRF image of an anterior pituitary cell. Several GFP-TSH granules that disappeared within 5 s are circled in red. Scale bar, 20 µm. (D) Number of expelled TSH granules were morphologically identified using the TIRF images (*n* = 5). Statistical analyses were performed using Fisher's PLSD test. *P<0.05 versus control. Values represent mean±SEM (% of control: B and D).

We next examined whether SIRT1 regulates TSH secretion by manipulating SIRT1 expression level in pituitary cells. SIRT1-expression plasmids or SIRT1-RNAi was introduced by electroporation into primary cultures of rat anterior pituitary. The cultured cells expressed either increased (overexpression) or decreased (knockdown) levels of SIRT1 protein compared to the controls (Fig. 2A). SIRT1 overexpression and knockdown led to an increase and decrease, respectively, in the amount of TSH secreted into the medium (Fig. 2B).

To test the effect of SIRT1 on the release of secretory granules, we used rat pituitary cells transfected with an expression vector encoding enhanced green fluorescent protein (EGFP) fused in frame with TSHβ. TSH is a 28k-Da heterodimer composed of covalently linked α and β subunits. The α subunit is common to TSH, FSH, and LH, whereas the β subunit is specific to the TSH molecule [26]. We observed, in real-time, the motion of individual EGFP-TSHβ granules undergoing exocytosis in living cells by using total internal reflection fluorescence (TIRF) microscopy—a method that enables fluorescence excitation within a closely restricted domain situated close to the plasma membrane [21], [27]. Given the characteristic of TIRF microscopy, the granule disappearance represents exocytosis of EGFP-TSHβ or returning of vesicles to cytoplasm via kiss-and-run recycling system. Thus the disappearance of granules is a result of vesicle trafficking. We recorded sequential images every 5 s (Video S1–S3) and counted the number of EGFP-positive granules that disappeared during the 5 s intervals (surrounded by red circles in Fig. 2C). The number of vanishing granules was increased in the SIRT1-overexpressing cells, and conversely decreased in the SIRT1-RNAi knockdown cells compared to the control cells (Fig. 2D). Hence, the trafficking of TSH-containing vesicles was more active in the SIRT1-overexpressing cells, and lesser in SIRT1-RNAi knockdown cells, compared to the control cells. These findings show that SIRT1 activates TSH release in thyrotropes.

### Identification of PIP5Kγ as a SIRT1-binding protein

**Figure 3:**
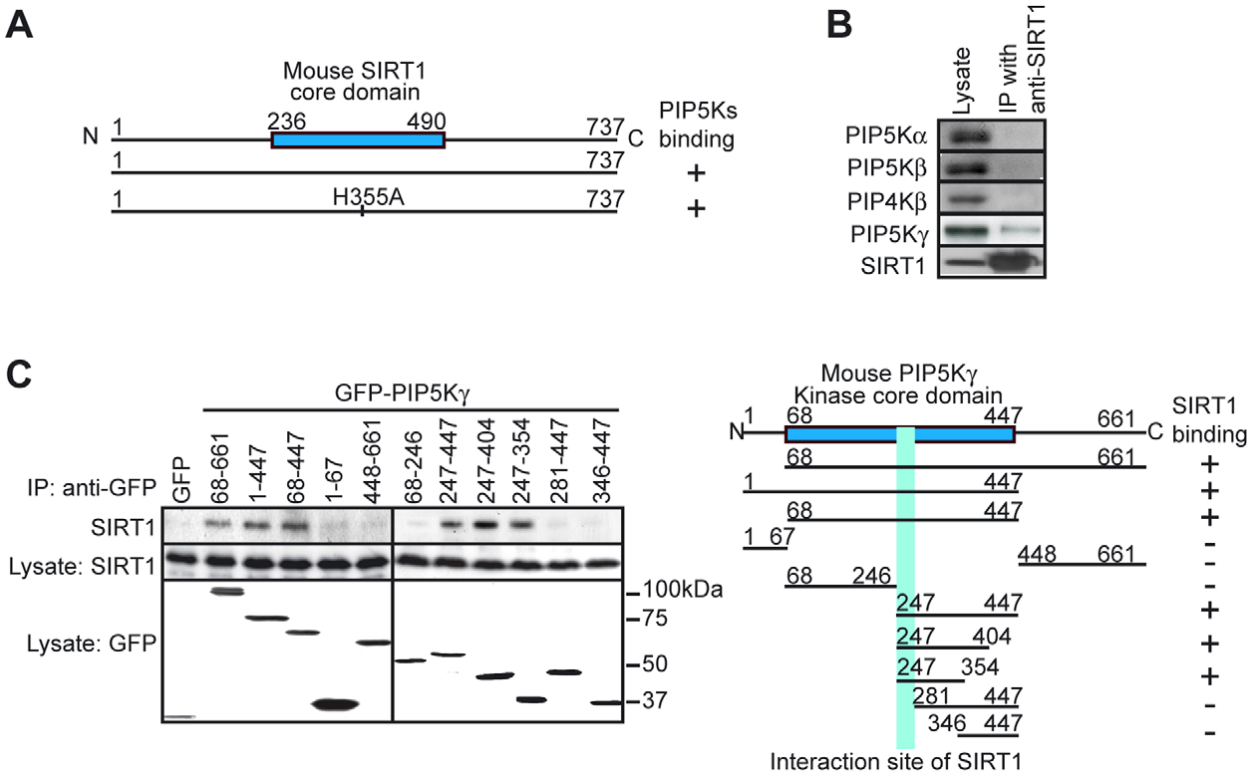
Identification of PIP5Kγ as a SIRT1-binding protein. (A) PIP5Ks were identified as SIRT1-associated protein by immunoprecipitation with anti-FLAG antibodies from HEK293T cells expressing FLAG-SIRT1. A full-length SIRT1 and inactive form of SIRT1 (H355A) interacted with PIP5K (+). (B) Immunoprecipitation analysis of endogenous SIRT1 and endogenous PIP5Kα, PIP5Kβ, PIP5Kγ, or PIP4Kβ in HEK293T cells. (C) Mapping the interaction interface of SIRT1 with PIP5Kγ. A schematic representation of the SIRT1 interaction site (right panel; positive (+), negative (−)).

To determine the molecular mechanisms that regulate TSH release by SIRT1, we sought out the potential targets that could physically associate with SIRT1. It is known that the catalytically inactive form of protein kinase binds more avidly to its substrates than the native form of enzyme, so substrates are more effectively pulled down with the mutant enzyme compared with the wild-type kinase [28]. Thus we used the catalytically inactive form (H355A) of SIRT1 [29], [30], to screen for binding partners. The Flag-tagged SIRT1 and SIRT1 (H355A) constructs were expressed in HEK293T cells and immunoprecipitated with an anti-Flag antibody. The immunoprecipitates were digested and then subjected to a nanoscale liquid chromatography-tandem mass spectrometry (LC-MS/MS) system [31]. Proteins identified as candidates of SIRT1 binding partners included phosphatidylinositol-4-phosphate 5-kinase (PIP5Ks) (Fig. 3A). The enzymes PIP5Ks (PIP5Kα, PIP5Kβ, and PIP5Kγ) are enzymes that catalyze the synthesis of PI(4,5)P_2_ mainly through the cellular route [32]. We focused on PIP5Ks since PIP5Kγ is reported to play an important role in the exocytosis of large dense-core vesicles by inducing the synthesis of PI(4,5)P_2_ in endocrine cells [33].

The common regions of all PIP5Ks were pulled down with SIRT1 in the fist screening with LC-MS/MS. To examine if SIRT1 more specially interacts with PIP5Kγ than with the other PIP5Ks, we performed immunoprecipitation analysis against endogenous proteins with antibodies to SIRT1 and to PIP5Ks. Amongst the PIP5Ks examined, PIP5Kγ was selectively immunoprecipitated with SIRT1 (Fig. 3B). Further investigation with deletion mutants of PIP5Kγ revealed that the interaction between SIRT1 and PIP5Kγ was mediated through a region of the kinase core domain [34] (amino acid residues 247—281) of PIP5Kγ (Fig. 3C).

### SIRT1-mediated deacetylation of PIP5Kγ

**Figure 4:**
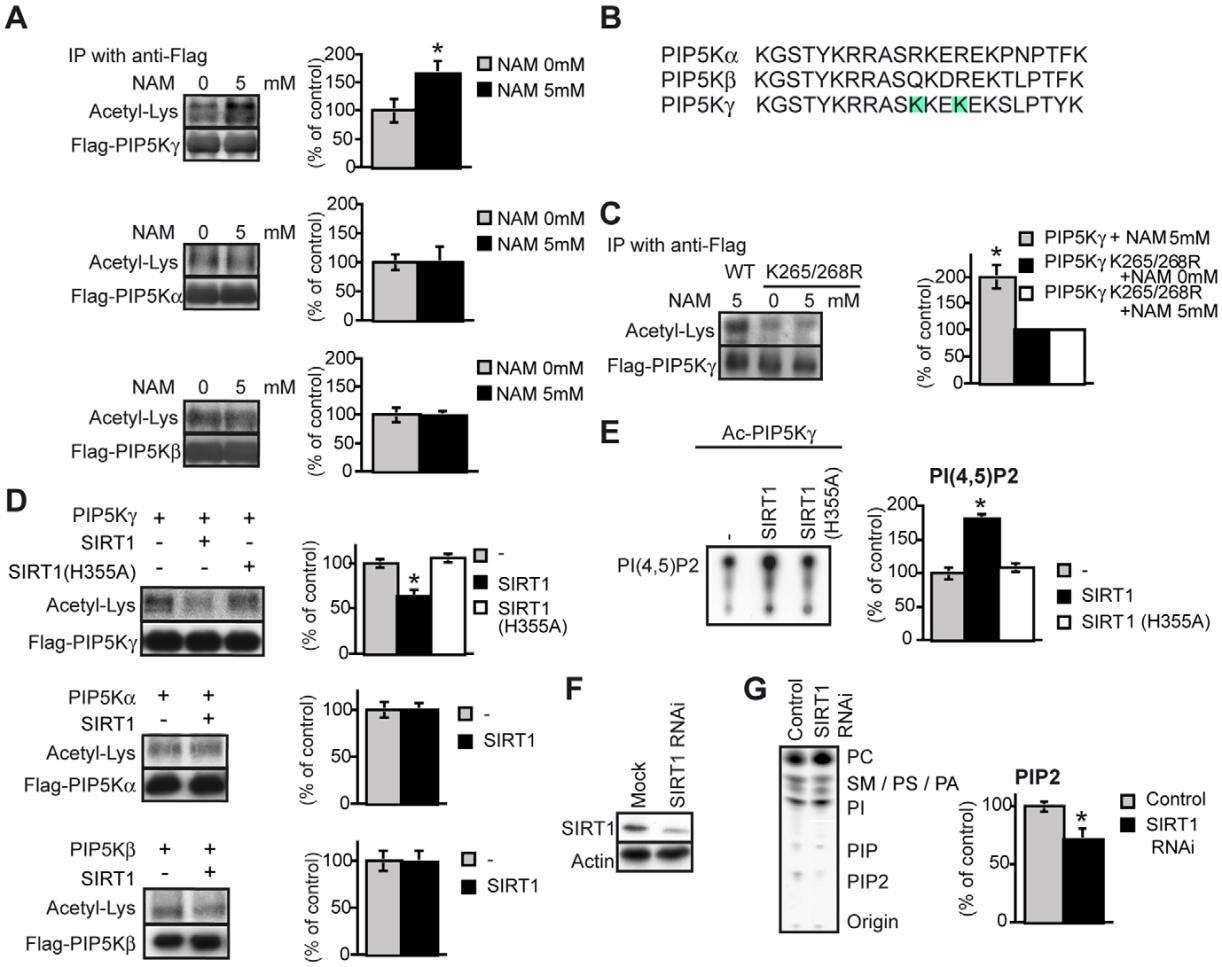
Activation of PIP5Kγ by deacetylation. (A, C) Effects of NAM on the acetylation of PIP5Ks (A) and PIP5Kγ K265/268R (C). The level of acetylated-lysine/PIP5K level was determined. Statistical analyses were performed using Student's *t* test (*n* = 3). *P<0.05 versus the non-NAM-treated group. (B) Sequence alignment of PIP5Ks. PIP5Kα (amino acids 205–226), PIP5Kβ (246–267), and PIP5Kγ (255–276) are shown. The light blue columns indicate candidates for deacetylation site by SIRT1. (D) *In vitro* deacetylation of PIP5Ks by SIRT1. Each Flag-PIP5Ks were purified from HEK293T cells after treatment with 5 mM NAM and was incubated with full-length recombinant SIRT1 or SIRT1 (H355A) in the presence of NAD^+^ for 3 h at 30°C. The acetylated-lysine/PIP5K level was determined. Statistical analyses were performed using Fisher's PLSD test or Student's *t* test (*n* = 3). *P<0.05 versus control. (E) TLC analysis of [^32^P]PI(4,5)P_2_ produced by acetylated PIP5Kγ incubated with recombinant SIRT1 or SIRT1 (H355A). The PI(4,5)P_2_ level was measured using the ImageJ software (*n* = 3). Statistical analyses were performed using Fisher's PLSD test. *P<0.05 versus control. (F) Western blot analysis of SIRT1 in HEK293 cells and SIRT1 RNAi cells. Actin was used as the control. (G) TLC analysis of [^32^P]PIP_2_ that was extracted from SIRT1 KD HEK 293T cells metabolically labelled (for 24 h) with [^32^P]orthophosphate. The PIP_2_ level was corrected for the PC level (*n* = 5). PC, phosphatidylcholine; SM, sphingomyelin; PS, phosphatidylserine; PA, phosphatidic acid; PI, phosphatidylinositol; and PIP, phosphatidylinositol phosphate. Values represent mean±SEM (A–D and F).

The binding of SIRT1 with PIP5Kγ suggests that SIRT1 deacetylates acetylated PIP5Kγ. Treatment with nicotinamide (NAM), a SIRT1 inhibitor [35], increased the lysine acetylation of PIP5Kγ, but did not alter the acetylation levels of PIP5Kα and PIP5Kβ (Fig. 4A).

The SIRT1-binding site of PIP5Kγ has 2 lysine residues (residues 265 and 268; shaded in Fig. 4B) besides the 5 identical lysine residues conserved in all the 3 PIP5Ks (residues 255, 260, 266, 270 and 276) (Fig. 4B) [34]. Within light of the preferential interaction between SIRT1 and PIP5Kγ, we hypothesized that SIRT1 mainly deacetylates lysines 265 and/or 268 of PIP5Kγ. To test this hypothesis, we constructed a PIP5Kγ plasmid in which 2 lysine residues were substituted with arginine residues (K265/268R). As expected, the K265/268R mutant was not further acetylated, regardless of treatment with NAM (Fig. 4C). This indicates that SIRT1 deacetylates the residues 265 and/or 268 of PIP5Kγ. To prove that SIRT1 directly deacetylates PIP5Kγ, we performed an *in vitro* deacetylase activity assay. PIP5Kγ deacetylation occurred upon the addition of recombinant wild-type SIRT1 but not the inactive form of SIRT1 (H355A) (Fig. 4D). In contrast, PIP5Kα and PIP5Kβ were not deacetylated by SIRT1 (Fig. 4D). Taken together, our findings indicate that SIRT1 selectively deacetylates PIP5Kγ among all the PIP5Ks.

We next asked whether the acetylation state affects the enzymatic activity of PIP5Kγ. We measured the *in vitro* kinase activity of PIP5Kγ with some modifications of the assay system of previous reports [36], [37]. We preincubated PIP5Kγ with recombinant wild-type SIRT1 or the inactive form of SIRT1 (H355A) for 3 h at 30°C, and then examined the activity of PIP5Kγ. The production of PIP_2_ was significantly increased when PIP5Kγ was deacetylated with wild-type SIRT1, whereas no increase occured when PIP5Kγ was incubated with the inactive SIRT1 (H355A) (Fig. 4E). These results indicate that SIRT1 deacetylates PIP5Kγ, thereby increasing the latter's activity. Consistent with this, PIP_2_ production was significantly decreased in HEK293T cells treated with SIRT1-RNAi (Fig. 4F and 4G). Our findings demonstrate that SIRT1 increases the kinase activity of PIP5Kγ by deacetylating this kinase.

### Involvement of PIP5Kγ in SIRT1-mediated TSH Secretion

**Figure 5:**
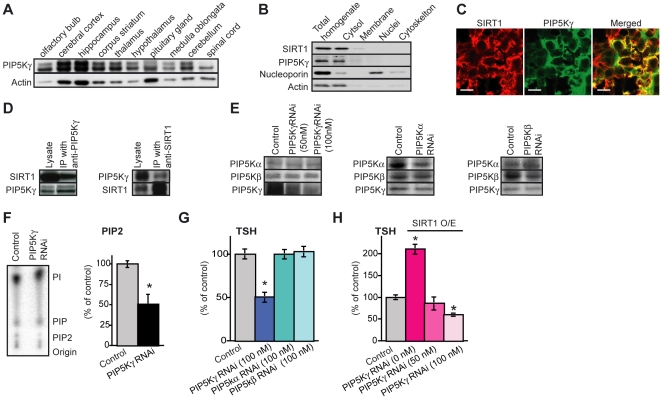
SIRT1 regulates TSH secretion via PIP5Kγ (K265/K268). (A) Western blot analysis of PIP5Kγ in the nerve tissues of mice. (B) Distribution of SIRT1 and PIP5Kγ in fractionated pituitary gland of mice. SIRT1 and PIP5Kγ were found to be present in cytosolic fractions rather than nuclear fractions. The first lane contains crude extract prior to separation. Nucleoporin, a nuclear marker; actin, a cytosolic fraction marker. (C) Double immunostaining with SIRT1 (red) and PIP5Kγ (green) in the anterior pituitary gland of rats. Scale bar, 10 mm. (D) Co-immunoprecipitation analysis of SIRT1 and PIP5Kγ in the cytosolic fraction of the pituitary gland of mice. Rabbit IgG was used as the negative control. (E) Western blot analysis of each PIP5K in PIP5Ks RNAi knockdown cells. (F) TLC analysis of [^32^P]PIP_2_ was extracted from PIP5Kγ RNAi pituitary cells metabolically labelled (for 24 h) with [^32^P]orthophosphate. The PIP_2_ level was corrected for the PC level (*n* = 5). (G) Amounts of TSH secreted in each PIP5Ks RNAi knockdown cells (*n* = 16). (H) Amounts of TSH secreted in cells overexpressing SIRT1 and PIP5Kγ RNAi (*n* = 16). Empty vectors and random RNAi constructs were used as controls. Statistical analyses were performed using Fisher's PLSD test. *P<0.05 versus control. Values represent mean±SEM (% of control: F–H).

PIP5Kγ is the major PI(4,5)P_2_ synthesizing enzyme in the brain [38]. In addition, PIP5Kγ KO mice show defects in vesicle trafficking [33]. We examined whether PIP5Kγ was expressed and physically interacted with SIRT1 in the pituitary gland, before testing whether PIP5Kγ is involved in the SIRT1-mediated TSH secretion in pituitary cells. PIP5Kγ is expressed in pituitary gland in addition to other regions of the mouse brain (Fig. 5A). In the pituitary gland, SIRT1 and PIP5Kγ were detected in the cytosolic fraction (Fig. 5B). Further, they colocalized in the cytoplasm of many cells in the anterior pituitary gland (Fig. 5C). These two proteins were coimmunoprecipitated with each other from pituitary cytosolic lysates by antibodies specific for each protein (Fig. 5D). Thus, SIRT1 and PIP5Kγ are physically associated in pituitary cells.

To determine whether PIP5Kγ is involved in SIRT1-mediated TSH secretion in pituitary cells, we knocked down PIP5Kγ in SIRT1-overexpressing pituitary cells. The specificities of RNAi against PIP5Ks were examined; RNAi against each PIP5K showed the specific knockdown effect to each target (Fig. 5E). We confirmed that PIP_2_ production was significantly decreased in the PIP5K-RNAi knockdown pituitary cells (Fig. 5F). Basal-level TSH secretion was reduced by the knockdown of PIP5Kγ, whereas neither PIP5Kα-RNAi nor PIP5Kβ-RNAi knockdown showed such an effect (Fig. 5G). Strikingly, the knockdown of PIP5Kγ completely abolished the effect of SIRT1 overexpression on the enhancement of TSH secretion (Fig. 5H). This result demonstrates that SIRT1 and PIP5Kγ work in the same pathway, and supports our concept that SIRT1 regulates TSH secretion via the deacetylation of PIP5Kγ.

### SIRT1 KO mice show high acetylated-PIP5Kγ, low PIP_2_ and low TSH Secretion

**Figure 6:**
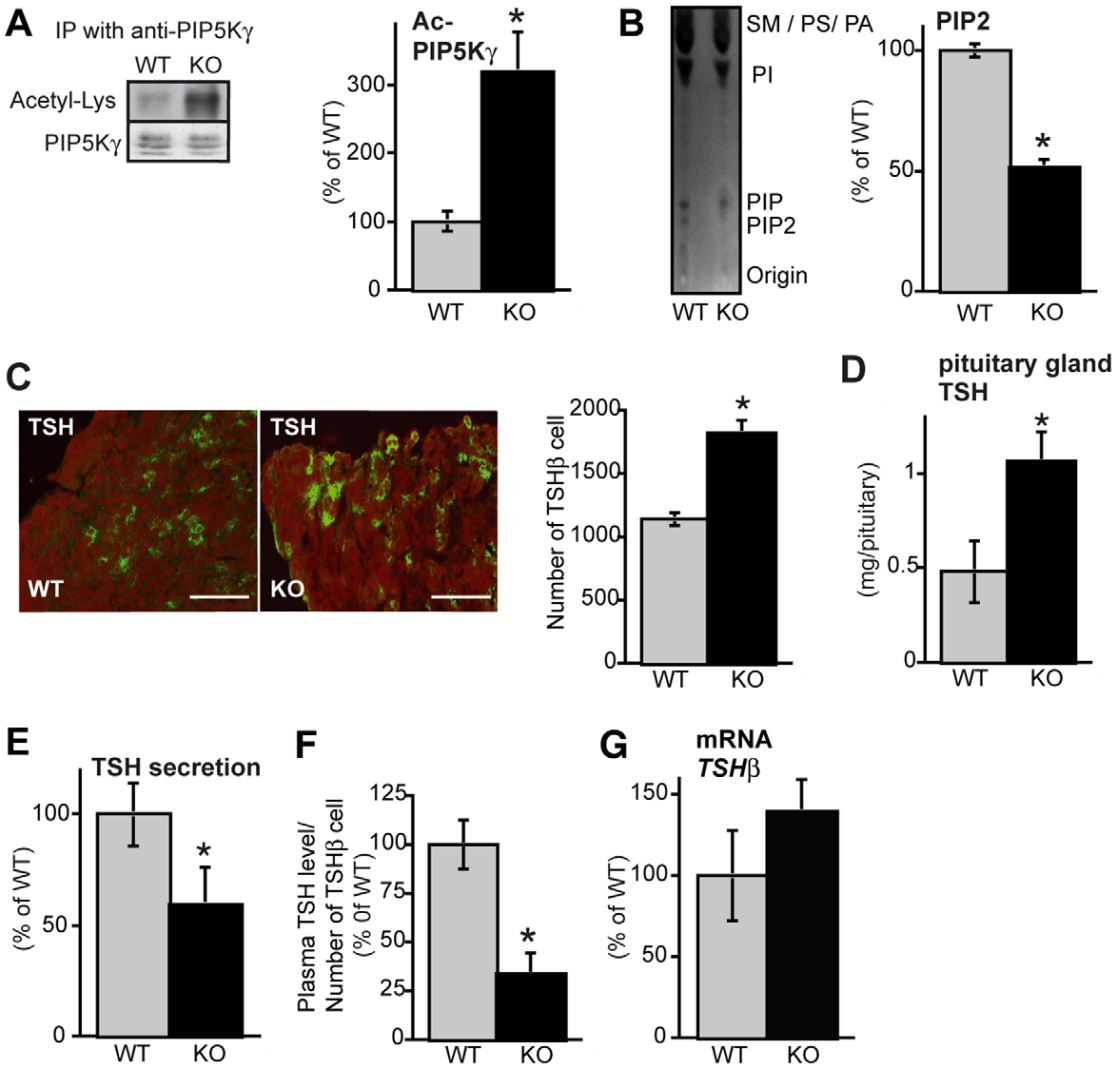
High Ac-PIP5Kγ, low PIP_2_, and low TSH secretion in pituitary glands obtained from SIRT1 KO mice. (A) Levels of acetylated PIP5Kγ in the pituitary of SIRT1 KO mice and WT mice. Endogenous PIP5Kγ was immunoprecipitated. The acetylated-lysine/PIP5Kγ level was measured (*n* = 3). (B) TLC analysis of PIP_2_ levels in the brain of SIRT1 KO mice and WT mice (WT: *n* = 6, KO: *n* = 6). (C) The number of TSHβ-immunopositive cells in SIRT1 KO mice and WT mice (*n* = 5). (D) Pituitary concentrations of TSH in SIRT1 KO mice and WT mice (*n* = 10). (E) Levels of TSH secreted by the pituitary cells in the organ culture over 24 h in SIRT1 KO mice and WT mice (*n* = 7). (F) Plasma TSH levels per TSH-immnunopositive cell in SIRT1 KO mice and WT mice (*n* = 5). (G) mRNA levels of anterior pituitary hormone in SIRT1 KO mice and WT mice. RNA levels were determined using RT-PCR analysis. GAPDH was used as a loading control (*n* = 3). Values represent mean±SEM. Statistical analyses were performed using Student's *t* test or Mann-Whitney *U* test. *P<0.05 versus WT.

To confirm that SIRT1 regulates TSH secretion *in vivo* via modulating PIP5Kγ acetylation level and its lipid kinase activity, we investigated SIRT1 KO mice. First, we examined the level of acetylated PIP5Kγ in the pituitary gland of SIRT1 KO mice. The level of acetylated PIP5Kγ was higher in SIRT1 KO mice than that in wild-type (WT) mice (Fig. 6A). The PIP_2_ levels in the brain were lower in the SIRT1 KO mice than in the WT mice (Fig. 6B).

**Figure 7:**
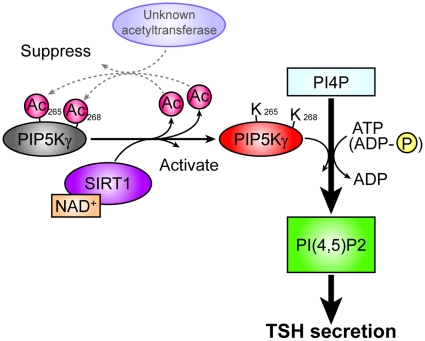
Model for the potential roles of SIRT1 in TSH secretion. Proposed model for regulation of TSH secretion by SIRT1.

SIRT1 KO mice have smaller pituitary than wild-type mice [39]. Thus, we examined the number of TSHβ cells in SIRT1 KO mice, before investigating the TSH release. Unexpectedly, the number of TSHβ cells was higher in the pituitary glands of SIRT1 KO when compared to normal (Fig. 6C). Consistent with this finding, the total TSH protein content in SIRT1 KO pituitary was significantly elevated (Fig. 6D). Despite the increased pituitary TSH contents, the amount of secreted TSH from SIRT1 KO pituitary glands was significantly lower than that from WT pituitary glands (Fig. 6E). The difference was more remarkable when the plasma TSH level was normalized with the number of pituitary TSHβ cells (Fig. 6F). There was no difference in the TSH mRNA levels in the thyrotropes of SIRT1 KO and WT mice (Fig. 6G). Taken together, these findings support our model that SIRT1 regulates TSH release through modulating PIP5Kγ activity (Fig. 7).

## Discussion

In the present study, we showed that SIRT1 is abundantly expressed in TSH-producing cells in the anterior pituitary gland. Hypothalamic–pituitary–thyroid (HPT) axis regulates energy expenditure, oxygen consumption, and fuel metabolism [40]. Thyroid hormones (triiodothyronine [T3] and thyroxine [T4]) negatively regulate the HPT axis [41], [42], and influence adipose tissue metabolism and cholesterol homeostasis [43]. In addition, the combination of serum TSH and tissue insulin sensitivity has important effects on serum lipid parameters in type 2 diabetes [44]. SIRT1 is also involved in insulin sensitivity and lipid metabolism [5]. Under fasting conditions, SIRT1 has a negative effect on insulin sensitivity [45]. Under fed conditions, activation of SIRT1 improves insulin sensitivity [46], [47]. Thus it is reasonable to speculate that the SIRT1-PIP5Kγ pathway described in this study represents a key event in the process of energy metabolism.

The longevity response to calorie restriction (CR) is actively regulated by nutrient-sensing pathways involving the kinase target of rapamycin (TOR) [48], AMP kinase [49], [50] and insulin/insulin-like growth factor (IGF-1) signalling [51] in lower organisms and mice. Sirtuins is also thought to be linked to CR and autophagy to prolong lifespan with TOR pathway [52]. Resveratrol, an activator of sirtuins, antagonizes the mTOR/S6K pathway [53]. In addition, sirtuins and TOR have opposing effects on autophagy independently from each other. Thus sirutins and the downstream signalling pathway form a complex network. It will be important to study to address whether SIRT1 in the pituitary also controls the other endocrine axes and other downstream target involving aging and lifespan.

In thyrotropes, SIRT1 was found to be distributed in the cytosol, suggesting a role of SIRT1 in the cytoplasm (Fig. 5B). In fact, we confirmed the colocalization and binding between PIP5Kγ and SIRT1 in the pituitary gland (Fig. 5C). SIRT1 has been shown to shuttle between the cytoplasm and nucleus [54]. SIRT1 is localized in cytoplasm of pancreatic a cells and endothelial cells, and localized in nuclei in liver, muscle, and white adipose tissue [9], [55]. Consistent with our findings that SIRT1 and PIP5Kγ were localized in the cytoplasm of pituitary gland cells, SIRT1 and endothelial nitric oxide synthase (eNOS) also colocalize and coprecipitate in cytoplasm of endothelial cells [55]. SIRT1 deacetylates eNOS for stimulating the activity and increases endothelial nitric oxide (NO) [55]. This result suggests SIRT1 has some targets in the cytoplasm as well as in the nucleus.

Our findings indicate that SIRT1 deacetylates acetylated PIP5Kγ at K265/K268, thereby activating TSH secretion from the pituitary gland (Fig. 7). The deacetylated form of PIP5Kγ is more enzymatically active and the PIP_2_ synthesized the secretion of TSH from pituitary thyrotropes. K265 and K268 of PIP5Kγ correspond to R215 and R218 of PIP5Kβ in human, respectively. The region from Y209 to R215 in PIP5Kβ is a phosphatidic acid (PA)-binding site, and the kinase activity of PIP5Kβ is completely lost when basic residues in this site are substituted with acidic residues [56]. This loss of function is thought to be caused by the alteration of the electrostatic interactions in the pocket composed of the PA-binding site and the surrounding residues. Since acetylation of lysine residues causes the alteration of electrical charge, the acetylation-deacetylation cycle we describe can easily be understood to regulate the kinase activity of PIP5Kγ. Although our work points to SIRT1 as the deacetylase responsible for activating PIP5Kγ, we have no information of which protein acetyltransferase is involved in the inactivating (acetylation) reaction.

We also visualized the vesicle of EGFP-TSHβ on exocytosis in primary culture of pituitary cells by using TIRF microscopy. Because the TIRF microscope only visualizes vesicles just beneath the membrane, the traffic event is mainly reflecting exocytosis [27], [57], [58]. The number of disappeared TSH-containing granules within 5 s was increased in the SIRT1-overexpressing cells compared to the control cells (Fig. 2C and 2D), confirming the activation of vesicle trafficking. In accordance with our findings, previous work has shown that the absence of PIP5Kγ causes a reduction of the readily releasable vesicle pool and a delay of fusion pore expansion indicating a defect in vesicle priming in chromaffin cells [33]. Our assay system revealed that PIP5Kγ knockdown inhibited PIP_2_ production and TSH secretion (Fig. 5G and 5H). Produced PI(4,5)P2 produced by PIP5Kγ is an important regulator of synaptic vesicle trafficking in nerve terminal [59]. Although SIRT1 has a many substrates, the SIRT1-PIP5Kγ pathway is important for intracellular trafficking and secretion in the thyrotropes. We also visualized the vesicle of EGFP-growth hormone (GH) on exocytosis in GH3 pituitary cells by using as TIRF microscopy (data not shown). Consistent with the observation of TSH-containing granules trafficking, the numbers of disappearing GH-containing granules was increased in the SIRT1-overexpressing cells (data not shown). These findings showed that the SIRT1-PIP5Kγ secretion pathway is shared by thyrotropes and other pituitary endocrine cells.

The physiological properties of the SIRT1 KO mouse are also consistent with the model proposed here. The levels of PIP5Kγ acetylation were elevated in SIRT1 KO mice and the TSH secretion levels were lower (Fig. 6D). SIRT1 KO mice have smaller pituitary than wild-type mice, however differentiation of the anterior pituitary occurs normally [39]. GH levels and insulin-like growth factor (IGF)-1 are not affected in SIRT1 KO mice [39]. In neuron-specific SIRT1 KO mice, the production and secretion of GH but not other pituitary hormones is reduced [11]. The production of all pituitary hormones is regulated at least in part by signals from the hypothalamus. TSH-releasing hormone (TRH) secreted by the hypothalamus is the main stimulator of TSH synthesis and secretion [60], [61]. It is generally considered that low serum TSH levels are attributable to the inhibition of TSH synthesis in the pituitary, which depletes the existing reserves of the hormone (exhausting the TSH “stock”), without changing the rate of secretion (“flow”). However, in this study, SIRT1 regulates release (related to “flow”, not “stock”) of TSH, suggesting that the physiological regulation of the pituitary-thyroid axis is dependent on regulated secretion.

The activity of SIRT1 is thought to be modulated by the nutritional status of the cell [62]. Recently, the expression of SIRT1 regulates oncogenic viral protein HPV7 [63] and locally acting IGF-1 isoform (mIGF-1), not circulating IGF-1 isoform [64]. Thus the activity and expression of SIRT1 changes topically and temporally. The core circadian rhythm regulator protein circadian locomotor output cycles kaput (CLOCK) is a histone acetyltransferase, and its activity is counterbalanced by NAD^+^ and SIRT1 [65]. The circadian rhythm modulates metabolic activities on a long-term basis, while intracellular traffic regulation of TSH by SIRT1 might provide short-term control of metabolism dependent on nutrient availability.

## Materials and Methods

### Study subjects

SIRT1 KO mice were generated on a CD1 and 129/J mixed genetic background, and genotyped using a polymerase chain reaction (PCR)-based protocol [66]. Water and food was provided *ad libitum* for both groups. Human tissue sections were obtained from the Tokyo Medical and Dental University, and Tokyo Metropolitan Institute of Gerontology. These experiments were approved by the Mitsubishi Kagaku Institute of Life Science Ethics Committee. All experiments were reformed according to protocols approved by the Animal Care and Use Committee of Mitsubishi Kagaku Institute of Life Sciences.

### Antibodies

We used the following antibodies: anti-SIRT1, β-actin, and Flag-M2 (Sigma-Aldrich); PIP5Kγ and nucleoporin (BD Transduction Laboratories); acetylated lysine (BioVision); GFP and agarose-conjugated GFP (Medical & Biological Laboratories); GAPDH (Millipore, Bedford, MA); ACTH (Nichirei), FSH and GH (DakoCytomation); TOTO-3 (Invitrogen). The anti-TSH, anti-LH, and anti-PRL antibodies were gifted by the Biosignal Research Center, Institute for Molecular and Cellular Regulation, Gunma University. For immunoprecipitation, Kanaho's lab prepared polyclonal antibodies against mouse PIP5Kα, PIP5Kβ, and PIP5Kγ [37]. PIP4Kβ, prepared in Moses V. Chao's lab, was detected by using a polyclonal antibody [67]. Secondary antibodies were obtained from Jackson Immunoresearch and Molecular Probes.

### Protein identification by LC-MS/MS analysis

Flag-SIRT1-associated complexes were digested with *Achromobacter* protease I, and the resulting peptides were analyzed using a nanoscale LC-MS/MS system. The criteria for match acceptance have been reported previously [31].

### Plasmids and transfection

Plasmids encoding Flag-tagged SIRT1 were constructed using the pCMV-Tag2 or pCMV-Tag4 vectors (Stratagene). SIRT1 (H355A) was generated by mutating histidine 355 to alanine [29], [30]. Plasmids encoding GFP-PIP5Ks or N-terminally epitope Flag-tagged PIP5Ks were constructed using the pcDNA3-GFP or pcDNA3-Flag (Invitrogen) vectors, as described previously [36]. The QuickChange Site-Directed Mutagenesis (Stratagene) kit was used to generate the PIP5Kγ (K265/268R) mutants. HEK293T cells were cultured in Dulbecco's modified Eagle's medium (DMEM) supplemented with 10% fetal calf serum. SIRT1 and PIP5K stealth RNAi were purchased from Invitrogen, and transfections were performed using Lipofectamine 2000 (Invitrogen) in accordance with the manufacturer's instructions. Rat TSHβ cDNA was generated by PCR. The PCR product was inserted into TagGFP (Evrogen). All constructs were confirmed by sequencing. The expression of TSHβ was confirmed by ELISA (Shibayagi), using lysates of GFP-TSHβ-overexpressing cells.

### Immunoprecipitation

The transfected HEK293T cells (6-cm dish) were lysed in lysis buffer (25 mM Tris-HCl [pH 8.0], 100 mM NaCl, 2 mM ethylenediamine acetic acid (EDTA) and 0.2% Triton X-100) containing protease inhibitors, 0.5 µM trichostatin A, and 5 mM NAM. After brief sonication, the lysates were cleared by centrifugation. The immunoprecipitates thus obtained were analyzed by immunoblotting. Cytoplasmic proteins obtained from rat pituitary were prepared using NE-PER Nuclear and Cytoplasmic Extraction Reagents, according to the manufacturer's instructions (Pierce).

### Western blot analysis

Lysate proteins were separated on an 8% SDS-PAGE gel and then blotted onto polyvinylidene difluoride membranes (Millipore). The membranes were blocked using Block Ace (Dainippon Seiyaku) and probed with the appropriate primary antibodies. The bound primary antibodies were detected using the corresponding horseradish peroxidase-conjugated secondary antibodies. The signal was visualized using the ECL kit (GE Healthcare Bio-Sciences). The signals were quantified using the ImageJ imaging software.

### Primary pituitary culture, electroporation and hormone assay

Pituitary cell cultures were prepared from the anterior pituitary glands of 12- to 15-week-old male Wistar-Imamichi rats. The pituitary glands were finely minced and then digested by rapid agitation in DMEM containing 4 mg/mL collagenase (type 2; Worthington), 2 mM L-glutamine, 25 mM HEPES, and 400 mg/mL DNase for 30 min at 37°C [68]. The pituitary cells were transfected using a microporator (Digital Bio Technology). In all, 1.5×10^5^ cells were electroporated with DNA (0.5 µg) or RNA (100 nM) at 1,500 V and a pulse width of 30 ms and seeded in 24-well plates (hormone assay) or 35-mm dishes (TIRF analysis). The cells were incubated in high-glucose DMEM containing 5% FBS and 15% horse serum at 37°C under 5% CO_2_. At 2 days after electroporation, the pituitary cells were preincubated in a buffer containing low amounts of K^+^ (140 mM NaCl, 4.7 mM KCl, 2.5 mM CaCl_2_, 1.2 mM MgSO_4_, 1.2 mM KH_2_PO_4_, 20 mM HEPES [pH 7.4] and 11 mM glucose) for 30 min, and then incubated in the same buffer for 24 h at 37°C. The levels of the hormones released into the culture medium were measured using radioimmunoassay kits for rat TSH (Shibayagi). The cells were harvested using celLytic buffer M (Sigma-Aldrich) containing protease inhibitors (Complete EDTA free; Roche Diagnostics). After removal of the cellular debris, the protein concentrations in the lysates were determined using the Bradford method. The pituitary cells were observed by TIRF confocal microscopy 1 day after electroporation. The plasma concentrations of mouse TSH were measured using radioimmunoassay kits (National Institute of Diabetes and Digestive and Kidney Diseases).

### TIRF microscopy

For TIRF microscopy, a total internal reflection system (Nikon) was used with minor modifications. Light from an Ar laser (488 nm) was introduced into an inverted epifluorescence microscope through a single-mode fiber and a double-illumination lens. The cells were observed in the thermostat-controlled stage (37°C).

### PIP5K acetylation assay and *in Vitro* deacetylation assay

HEK293T cells were transfected with Flag-PIP5Kγ, Flag-PIP5Kγ (K265/268R), Flag-PIP5Kα, or Flag-PIP5Kβ and treated with 0 mM or 5 mM NAM (Sigma-Aldrich). After 24 h, the cells were placed in celLytic buffer M and immunoprecipitated. The Flag-PIP5Ks were eluted using the Flag peptide. The eluted Flag-PIP5Ks were combined with either recombinant SIRT1 or SIRT1 (H355A) in a reaction buffer (50 mM Tris-HCl [pH 8.8], 4 mM MgCl_2_ and 0.5 mM DTT), 100 µM of NAD^+^ was added, and the complexes were incubated for 3 h at 30°C as described [30]. The reaction mixture was used for measuring the PIP5Kγ activity.

### Measurement of PIP5Kγ activity

The PIP5Kγ activity was determined according to the procedure described in a previously reported [36]. Briefly, 0.1 pmol of PIP5Kγ was incubated at 37°C for 25 min in 50 mL of 50 mM Tris–HCl, (pH 7.5), 1 mM EGTA, 10 mM MgCl_2_ and 0.004% (w/v) NP-40 containing 50 µM PI(4)P and 50 mM [γ-^32^P]ATP (0.1 µCi/assay). After extraction using the Bligh and Dyer method, the lipids were separated by thin-layer chromatography (TLC) as previously reported [36]. The [^32^P]PI(4,5)P_2_ produced was analyzed with an FLA2000 Bio-imaging analyzer (Fuji Photo Film).

### Lipid biochemistry

The HEK293T cells or pituitary cells were spread on a 6-cm dish at concentrations of 5×10^5^ cells/well and the plates were incubated for 24 h. These cells were transfected or electroporated with SIRT1 stealth RNAi incubated with 4mCi of [^32^P]orthophosphate in phosphate-free DMEM. After 48 h of incubation, the lipids were extracted using chloroform∶methanol∶1N HCl (3∶3∶1, v/v). The lipids were dissolved in chloroform∶methanol (2∶1) before being spotted onto a TLC plate (silica gel 60; Merck). The plate had been sprayed with potassium oxalate, using a solvent system comprising 1.0% potassium oxalate in methanol∶water (2∶3, v/v), and pre-activated by heating at 110°C for 60 min before the spotting. The chromatogram was developed in chloroform∶methanol∶20% methylamine (60∶36∶10, v/v/v). The radioactivity was visualized using a Fuji FLA2000 bioimaging analyzer. Lipids were extracted from the mouse brain, corrected for tissue weight, and TLC was performed as described above. Each spot was visualized using 0.005% primuline, a fluorescent dye. The signals were quantified using ImageJ imaging software.

### Immunohistochemistry

The isolated mouse and rat organs and the cultured cells were fixed with 4% paraformaldehyde and processed for immunostaining as described previously [69], [70]. Human pituitary glands were obtained by concluding autopsies on the bodies of 20 individuals aged 20—103 years at the time of death. None of these individuals had a clinical history of abnormal endocrine dysfunction. Half of each pituitary gland was serially sectioned and the sections were scanned at a magnification of 40Χ. The total number of cells and the number of TSH-immunopositive cells in the entire area in each field-of-view were counted [71].

### RT-PCR

Total RNA was extracted using Sepasol reagent (Nakalai Tesque), precipitated by ethanol in the presence of ethachinmate (Nippon Gene), and reverse transcribed using ReverTraAce (TOYOBO). We used the following primers: 5′-CGTCCCGTAGACAAAATGGT-3′ and 5′-GAATTTGCCGTGAGTGGAGT-3′ for mouse GAPDH, 5′-CGCATACGAGTGGAGAGAAA-3′ and 5′-ATGGCGACAGGGAAGGAGAA-3′ for mouse TSHb, 5′-CAGCCTGATGTTTGGTACCTCGGA-3′. Signals were quantified using ImageJ software.

### Statistical analysis

All the results are expressed as mean±SEM. The statistical significance of the differences between the groups was examined using Student's *t*-test, Mann-Whitney *U* test, or repeated-measures analysis of variance (ANOVA), as appropriate, and the STATVIEW program. When a significant F ratio was obtained, we conducted post-hoc analysis by using Fisher's protected least-significant difference (PLSD) test. P<0.05 was considered to be significant.

## Supporting Information

Video S1 Real-time motion of EGFP-TSHβ granules close to the plasma membrane of the pituitary cells was monitored at 5 s intervals by TIRF microscopy. Scale bar, 20 µm.
(0.06 MB AVI)

Video S2 Real-time motion of EGFP-TSHβ granules close to the plasma membrane of the SIRT1-overexpressing pituitary cells was monitored at 5 s intervals by TIRF microscopy. Scale bar, 20 µm.
(0.06 MB AVI)

Video S3 Real-time motion of EGFP-TSHβ granules close to the plasma membrane of SIRT1-RNAi pituitary cells was monitored at 5 s intervals by TIRF microscopy. Scale bar, 20 µm.
(0.06 MB AVI)

## References

[1] Imai S, Armstrong CM, Kaeberlein M, Guarente L (2000). Transcriptional silencing and longevity protein Sir2 is an NAD-dependent histone deacetylase.. Nature.

[2] North BJ, Verdin E (2004). Sirtuins: Sir2-related NAD-dependent protein deacetylases.. Genome Biol.

[3] Oberdoerffer P, Sinclair DA (2007). The role of nuclear architecture in genomic instability and ageing.. Nat Rev Mol Cell Biol.

[4] Tissenbaum HA, Guarente L (2001). Increased dosage of a sir-2 gene extends lifespan in Caenorhabditis elegans.. Nature.

[5] Finkel T, Deng CX, Mostoslavsky R (2009). Recent progress in the biology and physiology of sirtuins.. Nature.

[6] Picard F, Kurtev M, Chung N, Topark-Ngarm A, Senawong T (2004). Sirt1 promotes fat mobilization in white adipocytes by repressing PPAR-gamma.. Nature.

[7] Kahyo T, Mostoslavsky R, Goto M, Setou M (2008). Sirtuin-mediated deacetylation pathway stabilizes Werner syndrome protein.. FEBS Lett.

[8] Li K, Casta A, Wang R, Lozada E, Fan W (2008). Regulation of WRN protein cellular localization and enzymatic activities by SIRT1-mediated deacetylation.. J Biol Chem.

[9] Moynihan KA, Grimm AA, Plueger MM, Bernal-Mizrachi E, Ford E (2005). Increased dosage of mammalian Sir2 in pancreatic beta cells enhances glucose-stimulated insulin secretion in mice.. Cell Metab.

[10] Bordone L, Motta MC, Picard F, Robinson A, Jhala US (2006). Sirt1 regulates insulin secretion by repressing UCP2 in pancreatic beta cells.. PLoS Biol.

[11] Cohen DE, Supinski AM, Bonkowski MS, Donmez G, Guarente LP (2009). Neuronal SIRT1 regulates endocrine and behavioral responses to calorie restriction.. Genes & Development.

[12] Bordone L, Cohen D, Robinson A, Motta MC, van Veen E (2007). SIRT1 transgenic mice show phenotypes resembling calorie restriction.. Aging Cell.

[13] Ramadori G, Lee CE, Bookout AL, Lee S, Williams KW (2008). Brain SIRT1: anatomical distribution and regulation by energy availability.. J Neurosci.

[14] Cakir I, Perello M, Lansari O, Messier NJ, Vaslet CA (2009). Hypothalamic Sirt1 regulates food intake in a rodent model system.. PLoS ONE.

[15] Setou M, Nakagawa T, Seog DH, Hirokawa N (2000). Kinesin superfamily motor protein KIF17 and mLin-10 in NMDA receptor-containing vesicle transport.. Science.

[16] Setou M, Seog DH, Tanaka Y, Kanai Y, Takei Y (2002). Glutamate-receptor-interacting protein GRIP1 directly steers kinesin to dendrites.. Nature.

[17] Konishi Y, Setou M (2009). Tubulin tyrosination navigates the kinesin-1 motor domain to axons.. Nat Neurosci.

[18] Ikegami K, Heier RL, Taruishi M, Takagi H, Mukai M (2007). Loss of alpha-tubulin polyglutamylation in ROSA22 mice is associated with abnormal targeting of KIF1A and modulated synaptic function.. Proc Natl Acad Sci U S A.

[19] Yao I, Takagi H, Ageta H, Kahyo T, Sato S (2007). SCRAPPER-Dependent Ubiquitination of Active Zone Protein RIM1 Regulates Synaptic Vesicle Release.. Cell.

[20] Matsuno A, Itoh J, Mizutani A, Takekoshi S, Osamura RY (2008). Co-transfection of EYFP-GH and ECFP-rab3B in an experimental pituitary GH3 cell: a role of rab3B in secretion of GH through porosome.. Folia Histochem Cytobiol.

[21] Matsuno A, Mizutani A, Itoh J, Takekoshi S, Nagashima T (2005). Establishment of stable GH3 cell line expressing enhanced yellow fluorescein protein-growth hormone fusion protein.. J Histochem Cytochem.

[22] De Camilli P, Emr SD, McPherson PS, Novick P (1996). Phosphoinositides as regulators in membrane traffic.. Science.

[23] Bai J, Tucker WC, Chapman ER (2004). PIP2 increases the speed of response of synaptotagmin and steers its membrane-penetration activity toward the plasma membrane.. Nat Struct Mol Biol.

[24] Boily G, Seifert EL, Bevilacqua L, He XH, Sabourin G (2008). SirT1 regulates energy metabolism and response to caloric restriction in mice.. PLoS ONE.

[25] Dorshkind K, Horseman ND (2001). Anterior pituitary hormones, stress, and immune system homeostasis.. Bioessays.

[26] Thotakura NR, Blithe DL (1995). Glycoprotein hormones: glycobiology of gonadotrophins, thyrotrophin and free alpha subunit.. Glycobiology.

[27] Letinic K, Sebastian R, Toomre D, Rakic P (2009). Exocyst is involved in polarized cell migration and cerebral cortical development.. Proc Natl Acad Sci U S A.

[28] Swayze RD, Braun AP (2001). A catalytically inactive mutant of type I cGMP-dependent protein kinase prevents enhancement of large conductance, calcium-sensitive K+ channels by sodium nitroprusside and cGMP.. J Biol Chem.

[29] Luo J, Nikolaev AY, Imai S, Chen D, Su F (2001). Negative control of p53 by Sir2alpha promotes cell survival under stress.. Cell.

[30] Rodgers JT, Lerin C, Haas W, Gygi SP, Spiegelman BM (2005). Nutrient control of glucose homeostasis through a complex of PGC-1alpha and SIRT1.. Nature.

[31] Natsume T, Yamauchi Y, Nakayama H, Shinkawa T, Yanagida M (2002). A direct nanoflow liquid chromatography-tandem mass spectrometry system for interaction proteomics.. Anal Chem.

[32] Stephens LR, Hughes KT, Irvine RF (1991). Pathway of phosphatidylinositol(3,4,5)-trisphosphate synthesis in activated neutrophils.. Nature.

[33] Gong LW, Di Paolo G, Diaz E, Cestra G, Diaz ME (2005). Phosphatidylinositol phosphate kinase type I gamma regulates dynamics of large dense-core vesicle fusion.. Proc Natl Acad Sci U S A.

[34] Ishihara H, Shibasaki Y, Kizuki N, Wada T, Yazaki Y (1998). Type I phosphatidylinositol-4-phosphate 5-kinases. Cloning of the third isoform and deletion/substitution analysis of members of this novel lipid kinase family.. J Biol Chem.

[35] Bitterman KJ, Anderson RM, Cohen HY, Latorre-Esteves M, Sinclair DA (2002). Inhibition of silencing and accelerated aging by nicotinamide, a putative negative regulator of yeast sir2 and human SIRT1.. J Biol Chem.

[36] Honda A, Nogami M, Yokozeki T, Yamazaki M, Nakamura H (1999). Phosphatidylinositol 4-phosphate 5-kinase alpha is a downstream effector of the small G protein ARF6 in membrane ruffle formation.. Cell.

[37] Nakano-Kobayashi A, Yamazaki M, Unoki T, Hongu T, Murata C (2007). Role of activation of PIP5Kgamma661 by AP-2 complex in synaptic vesicle endocytosis.. EMBO J.

[38] Wenk MR, Pellegrini L, Klenchin VA, Di Paolo G, Chang S (2001). PIP kinase Igamma is the major PI(4,5)P(2) synthesizing enzyme at the synapse.. Neuron.

[39] Lemieux ME, Yang X, Jardine K, He X, Jacobsen KX (2005). The Sirt1 deacetylase modulates the insulin-like growth factor signaling pathway in mammals.. Mech Ageing Dev.

[40] Lowell BB, Spiegelman BM (2000). Towards a molecular understanding of adaptive thermogenesis.. Nature.

[41] Abel ED, Ahima RS, Boers ME, Elmquist JK, Wondisford FE (2001). Critical role for thyroid hormone receptor beta2 in the regulation of paraventricular thyrotropin-releasing hormone neurons.. J Clin Invest.

[42] Weiss RE, Forrest D, Pohlenz J, Cua K, Curran T (1997). Thyrotropin regulation by thyroid hormone in thyroid hormone receptor beta-deficient mice.. Endocrinology.

[43] Shin DJ, Osborne TF (2003). Thyroid hormone regulation and cholesterol metabolism are connected through Sterol Regulatory Element-Binding Protein-2 (SREBP-2).. J Biol Chem.

[44] Chubb SA, Davis WA, Davis TM (2005). Interactions among thyroid function, insulin sensitivity, and serum lipid concentrations: the Fremantle diabetes study.. J Clin Endocrinol Metab.

[45] Rodgers JT, Puigserver P (2007). Fasting-dependent glucose and lipid metabolic response through hepatic sirtuin 1.. Proc Natl Acad Sci U S A.

[46] Sun C, Zhang F, Ge X, Yan T, Chen X (2007). SIRT1 improves insulin sensitivity under insulin-resistant conditions by repressing PTP1B.. Cell Metab.

[47] Milne JC, Lambert PD, Schenk S, Carney DP, Smith JJ (2007). Small molecule activators of SIRT1 as therapeutics for the treatment of type 2 diabetes.. Nature.

[48] Kenyon CJ (2010). The genetics of ageing.. Nature.

[49] Canto C, Gerhart-Hines Z, Feige JN, Lagouge M, Noriega L (2009). AMPK regulates energy expenditure by modulating NAD+ metabolism and SIRT1 activity.. Nature.

[50] Greer EL, Dowlatshahi D, Banko MR, Villen J, Hoang K (2007). An AMPK-FOXO pathway mediates longevity induced by a novel method of dietary restriction in C. elegans.. Curr Biol.

[51] Grandison RC, Piper MD, Partridge L (2009). Amino-acid imbalance explains extension of lifespan by dietary restriction in Drosophila.. Nature.

[52] Blagosklonny MV (2010). Linking calorie restriction to longevity through sirtuins and autophagy: any role for TOR.. Cell Death and Disease.

[53] Armour SM, Baur JA, Hsieh SN, Land-Bracha A, Thomas SM (2009). Inhibition of mammalian S6 kinase by resveratrol suppresses autophagy.. Aging.

[54] Tanno M, Sakamoto J, Miura T, Shimamoto K, Horio Y (2007). Nucleocytoplasmic shuttling of the NAD+-dependent histone deacetylase SIRT1.. J Biol Chem.

[55] Mattagajasingh I, Kim CS, Naqvi A, Yamamori T, Hoffman TA (2007). SIRT1 promotes endothelium-dependent vascular relaxation by activating endothelial nitric oxide synthase.. Proc Natl Acad Sci U S A.

[56] Stace C, Manifava M, Delon C, Coadwell J, Cockcroft S (2008). PA binding of phosphatidylinositol 4-phosphate 5-kinase.. Adv Enzyme Regul.

[57] James DJ, Khodthong C, Kowalchyk JA, Martin TF (2008). Phosphatidylinositol 4,5-bisphosphate regulates SNARE-dependent membrane fusion.. J Cell Biol.

[58] Zoncu R, Perera RM, Balkin DM, Pirruccello M, Toomre D (2009). A phosphoinositide switch controls the maturation and signaling properties of APPL endosomes.. Cell.

[59] Di Paolo G, Moskowitz HS, Gipson K, Wenk MR, Voronov S (2004). Impaired PtdIns(4,5)P2 synthesis in nerve terminals produces defects in synaptic vesicle trafficking.. Nature.

[60] Gary KA, Sevarino KA, Yarbrough GG, Prange AJ, Winokur A (2003). The thyrotropin-releasing hormone (TRH) hypothesis of homeostatic regulation: implications for TRH-based therapeutics.. J Pharmacol Exp Ther.

[61] Schally AV, Coy DH, Meyers CA (1978). Hypothalamic regulatory hormones.. Annu Rev Biochem.

[62] Leibiger IB, Berggren PO (2006). Sirt1: a metabolic master switch that modulates lifespan.. Nat Med.

[63] Allison SJ, Jiang M, Milner J (2009). Oncogenic viral protein HPV E7 up-regulates the SIRT1 longevity protein in human cervical cancer cells.. Aging.

[64] Vinciguerra M, Santini MP, Claycomb WC, Ladurner AG, Rosenthal N (2010). Local IGF-1 isoform protects cardiomyocytes from hypertrophic and oxidative stresses via SirT1 activity.. Aging.

[65] Nakahata Y, Kaluzova M, Grimaldi B, Sahar S, Hirayama J (2008). The NAD+-dependent deacetylase SIRT1 modulates CLOCK-mediated chromatin remodeling and circadian control.. Cell.

[66] McBurney MW, Yang X, Jardine K, Hixon M, Boekelheide K (2003). The mammalian SIR2alpha protein has a role in embryogenesis and gametogenesis.. Mol Cell Biol.

[67] Castellino AM, Parker GJ, Boronenkov IV, Anderson RA, Chao MV (1997). A novel interaction between the juxtamembrane region of the p55 tumor necrosis factor receptor and phosphatidylinositol-4-phosphate 5-kinase.. J Biol Chem.

[68] Renz M, Tomlinson E, Hultgren B, Levin N, Gu Q (2000). Quantitative expression analysis of genes regulated by both obesity and leptin reveals a regulatory loop between leptin and pituitary-derived ACTH.. J Biol Chem.

[69] Shindler KS, Roth KA (1996). Double immunofluorescent staining using two unconjugated primary antisera raised in the same species.. J Histochem Cytochem.

[70] Asai S, Ohta R, Fujikawa T, Sakai RR, Shirota M (2006). Gastric ulceration and expression of prolactin receptor in the brain in Hatano high- and low-avoidance rats.. Endocrine.

[71] Nikrodhanond AA, Ortiga-Carvalho TM, Shibusawa N, Hashimoto K, Liao XH (2006). Dominant role of thyrotropin-releasing hormone in the hypothalamic-pituitary-thyroid axis.. J Biol Chem.

